# Modifiable Prognostic Factors of Hepatocellular Carcinoma in Patients with Non-Surgical Treatment

**DOI:** 10.1371/journal.pone.0144893

**Published:** 2015-12-14

**Authors:** Jen-Hao Yeh, Chao-Hung Hung, Jing-Houng Wang, Chien-Hung Chen, Kwong-Ming Kee, Chung-Mou Kuo, Yi-Hao Yen, Yu-Fan Cheng, Yen-Yang Chen, Hsuan-Chi Hsu, Sheng-Nan Lu

**Affiliations:** 1 Division of Hepato-Gastroenterology, Department of Internal Medicine, Kaohsiung Chang Gung Memorial Hospital and Chang Gung University College of Medicine, Kaohsiung, Taiwan; 2 Department of Diagnostic Radiology, Kaohsiung Chang Gung Memorial Hospital, Kaohsiung, Taiwan; 3 Division of Hematology-Oncology, Department of Internal Medicine, Kaohsiung Chang Gung Memorial Hospital, Kaohsiung, Taiwan; 4 Department of Radiation Oncology, Kaohsiung Chang Gung Memorial Hospital, Kaohsiung, Taiwan; Taipei Veterans General Hospital, TAIWAN

## Abstract

**Background & aims:**

Current hepatocellular carcinoma (HCC) staging systems only use baseline characteristics to predict outcome. We aimed to explore modifiable factors of the prognosis in HCC cases had undergone non-surgical treatment.

**Methods:**

All HCC cases in Kaohsiung Chang Gung Memorial hospital in southern Taiwan from 2002 to 2012 must met all below criteria: (1) met international diagnostic guidelines, (2) underwent the initial treatments in our hospital (3) treated by non-surgical treatment modalities and (4) survived more than two years, with follow-up time longer than five years.

**Results:**

A total 698 patients were enrolled: 451 (24.6%, group A) survivied between 2 to 5 years, and 247 (13.5%, group B) had survived > 5 years. Aside from liver function reserve and BCLC stages, four interventional factors: initial treatment modality, outcomes of 1^st^ or 2^nd^ treatment, and anti-viral therapy to chronic viral hepatitis were associated with prognosis. After propensity score matching, multiple logistic regression of 223 well-matched pairs showed that recurrence within one year after 1^st^ treatment (OR: 2.17, 95% CI: 1.35–3.48), incomplete 2^nd^ treatment (2.01, 1.27–3.17) and absence of anti-viral agents (1.68, 1.09–2.59) were independent poor prognostic factors.

**Conclusion:**

Complete treatment and anti-viral agents to chronic hepatitis were both independent modifiable prognostic factors of HCC patients had undergone non-surgical treatment. Based on these findings, timely treatment to achieve maximal locoregional control and anti-viral treatment should be provided as possible.

## Introduction

HCC is an aggressive tumor with many staging system available for prognosis evaluation. The tumor, metastasis, and node staging has been used in surgical eligible HCC with convincible success [1]. However, many patients with HCC have not been surgical candidates, either due to advanced tumor extension or poor liver function reserve, therefore many other staging systems were developed, such as the Okuda system [2], the Cancer of the Liver Italian Program [3], the Barcelona clinic liver cancer (BCLC) [4] system, and the albumin-bilirubin grade [5]. While these systems differs in details, their performances are similar, as the Second Consensus Conference by the American Hepatico-Pancreatico-Biliary Association and American Joint Committee on Cancer in 2010 concluded that no single staging system is applicable to all patients with HCC [6]. On the other hand, parameters in these tools are exclusively baseline factors such as liver function reserve or initial tumor extent. In the past two decades, many non-surgical therapies have been developed and long-term survival or tumor free status can be sometimes achieved even without surgery [7–11]. During the treatment course, modifiable prognostic factors, if any, should be invaluable in guiding a proper treatment protocol, yet the relevant data is quite limited.

In this study, we aim to search for the modifiable prognostic factors of HCC in patients with initial non-surgical treatment.

## Material and Methods

### Patient selection

This is a single center, retrospective cohort study running from January 2002 to December 2012. Patients had newly diagnosed HCC, underwent non-surgical treatment modality in our hospital whose survival > 2 years were enrolled. The diagnosis of HCC adhered to current international guidelines [12, 13]; either by typical findings of dynamic computer tomography or magnetic resonance image, or biopsy. The exclusion criteria were: (1) prior HCC treatment, (2) subsequent liver resection or transplantation during follow-up, (3) best supportive care only, (4) less than five-year follow-up. The detailed patient stratification were illustrated at the supplement material (S1 Fig).

In all the patients, age, sex, status of viral hepatitis, Child-Pugh score, aspartate aminotransferase (AST), alanine aminotransferase (ALT), platelet count, albumin, bilirubin, alpha feto-protein (AFP), the Barcelona clinical liver cancer (BCLC) stage, status and treatment of viral hepatitis, and serial treatment modality were well-documented in the data base for further analysis.

### Definition and terminology

In this study, the treatment modality of HCC was a number of interventions aiming to achieve at least locoregional control or curative means, including radiofrequency ablation (RFA), percutaneous ethanol injection (PEI), transarterial embolization (TAE) with or without chemotherapy agent [14], radiation therapy (RT) or chemotherapy (CT). Sorafenib was not included due to its being approved in 2012 in Taiwan.

Initial treatment refers to the first treatment modality to the patient, based on BCLC stage, liver function reserve, preference of the patient, and clinical judgments. If more than one kind of treatment modality was performed within the same month, we used the following priority for classification: (1) RFA, (2) PEI, (3) TAE, and then (4) RT or CT. When multiple procedures were employed concurrently, we generally recognized the procedure with highest priority as the main treatment modality. For example, when one received both RFA and TAE as first treatment, RFA would be recognized as the initial treatment at record and subsequent analysis. However, in patients underwent PEI and TAE concurrently, they were classified as receiving PEI if it was done with curative intention; otherwise they were referred to TAE group when PEI only served as adjuvant management, as shown by image and chart review.

After the first treatment, a dynamic computer tomography or magnetic resonance image was performed one month later to evaluate treatment response. For this, we used the standardized terminology of the Interventional Working Group on Image-Guided Tumor Ablation to define technical effectiveness, in which complete treatment was defined in the absence of persistent enhancing tumor in the previous target foci or any other part; whereas incomplete treatment referred to any residual, irregularly enhanced tumors [15]. When complete treatment was documented, we performed liver ultrasonography and serum AFP measurement with/without a dynamic computer tomography/magnetic resonance image every three months.

After complete treatment, recurrence was defined as any new tumor > 1cm found during the follow-up period, either intrahepatic or extrahepatic that met diagnostic criteria by current international guidelines [12, 13]. If there were a patient with incomplete treatment, or documented recurrence after the first complete treatment, we offered secondary treatment, chosen from the afore-mentioned five interventional modalities based on tumor extent, liver reserve and performance status, as possible. The post-treatment evaluation and surveillance, and definition of recurrence were all identical to the first treatment. Of note, in patients that received RFA as first or secondary treatment, despite residual tumor existence in the one-month period, it could be considered a complete treatment if an immediate additional RFA resulted in radiologic tumor-free status in the next surveillance [16].

Viral hepatitis refers to those who were positive for hepatitis B surface antigen or anti-hepatitis C virus antibody at any time before or after diagnosis of HCC. With hepatitis B, treatment was defined as more than a one month course of any nucleotide/nucleotide analog (lamivudine, telbivudine, entecavir, adefovir or tenofovir), in either single or combination regimen; and/or at least one week of standard or pegylated interferon-alpha use. For hepatitis C, treatment was defined as at least one week of standard or pegylated interferon-alpha use with or without ribavirin, regardless of virological response. The anti-viral agent could be given before or after treatment of HCC, but part of the treatment course should have taken place in our hospital that fulfilled the afore-mentioned definition.

### Statistics

Statistical significance is considered when the *p* value is less than 0.05. Continuous variables were expressed as means +/- standard deviations and categorical variables were expressed as absolute and relative frequencies. We used an independent-samples t test to compare continuous data, and a chi-square test to compare categorical variables. Survival rates were obtained with the Kaplan-Meier method, and the survival curves among groups were illustrated at the supplement material (S2 Fig).

Propensity scoring was also used for control of selection bias and performed using binary logistic regression to generate a propensity score for each patient who underwent group (A) and (B). Variables included in the propensity model were gender, age, PT, HBsAg, anti-HCV, bilirubin, presence of ascites, encephalopathy, Child-Pugh, AST, ALT, platelet count, albumin, and AFP levels. BCLC stage was not included due to asymmetrical distributions of patients in each stage between both groups. After amending these confounding factors, simple and multiple binary logistic regression was used in evaluation of the interventional prognostic factors.

### Ethics

We established and managed the database while conforming to current Taiwanese legislation on privacy and clinical study. The study was approved by the local institutional review board in Chang Gung Memorial Hospital, and informed consent was not required for the retrospective medical records analysis. Besides, the patient records/information was anonymized and de-identified prior to analysis for this retrospective study.

## Results

### Baseline characteristics

698 patients were eligible patients in our study, and they were subdivided by the survival time: 451 survived two to five years (64.6%, group A), and 247 survived more than five years (35.4%, group B). Subsequent analysis revealed group B had better liver function profiles marked by higher proportion of normal serum albumin and bilirubin levels, lower prevalence of ascites, and less proportion of Child-Pugh score B-C (30.2% in group A and 9.3% in group B, *p*< 0.001); on the other hand, patients in group A tended to have more advanced BCLC stage upon diagnosis (Table 1).

**Table 1:** Baseline characteristics upon first treatment in Group A and B before and after propensity score match.

		Group A(n = 451,64.6%)	Group B(n = 247,35.4%)	*P* value	MatchedGroup A(n = 223)	MatchedGroup B(n = 223)	*P* value
Age	Years±SD	64.0±11.1	62.4±10.3	0.061	62.7±10.4	62.9±10.4	0.803
Gender	Male	320 (71.0%)	169 (68.4%)	0.485	153 (68.6%)	153 (68.6%)	1.000
HBsAg	Positive	188 (41.7%)	102 (41.3%)	0.920	94 (42.2%)	88 (39.5%)	0.563
Anti-HCV Ab	Positive	244 (54.1%)	133 (53.8%)	0.948	124 (55.6%)	124 (55.6%)	1.000
AST (IU/ml)	>40	346 (76.7%)	174 (70.4%)	0.069	154 (69.1%)	157 (70.4%)	0.757
ALT (IU/ml)	>40	295 (65.4%)	167 (67.6%)	0.557	140 (62.8%)	147 (65.9%)	0.485
Albumin (g/dl)	>3.5	191 (42.4%)	138 (55.9%)	*0.001	112 (50.2%)	118 (52.9%)	0.570
Bilirubin (mg/dl)	<2	381 (84.5%)	242 (98.0%)	*<0.001	219 (98.2%)	218 (97.8%)	0.736
PT prolong (sec)	< = 3	438 (97.1%)	244 (98.8%)	0.159	218 (97.8%)	220 (98.7%)	0.476
Ascites	None	381 (15.5%)	237 (96.0%)	*<0.001	211 (94.6%)	214 (96.0%)	0.502
Encephalopathy	None	448 (99.3%)	242 (98.0%)	0.107	222 (99.6%)	220 (98.7%)	0.315
Child-Pugh	A	315 (69.8%)	224 (90.7%)	*<0.001	196 (87.9%)	201 (90.1%)	0.449
	B	129 (28.6%)	23 (9.3%)		27 (12.1%)	22 (9.9%)	
	C	7 (1.6%)	0 (0)		0	0	
Platelet (/10^9^)	≤150	305 (67.6%)	155 (62.8%)	0.194	145 (65.0%)	144 (64.6%)	0.921
AFP (ng/ml)	<20	218 (48.3%)	152 (61.5%)	*0.003	125 (56.1%)	133 (59.6%)	0.730
	20–400	171 (37.9%)	74 (30.0%)		75 (33.6%)	70 (31.4%)	
	>400	62 (13.7%)	21 (8.5%)		23 (10.3%)	20 (9.0%)	
BCLC staging	Very early	40 (8.9%)	67 (27.1%)	*<0.001	37 (16.6%)	48 (21.5%)	0.472
	Early	209 (46.3%)	123 (49.8%)		130 (58.3%)	118 (52.9%)	
	Intermediate	140 (31.0%)	46 (18.6%)		48 (21.5%)	46 (20.6%)	
	Advanced	55 (12.2%)	11 (4.5%)		8 (3.6%)	11 (4.9%)	
	Terminal	7 (1.6%)	0 (0%)		0	0	

*:p< 0.01

### Initial treatment and response

**Fig 1:**
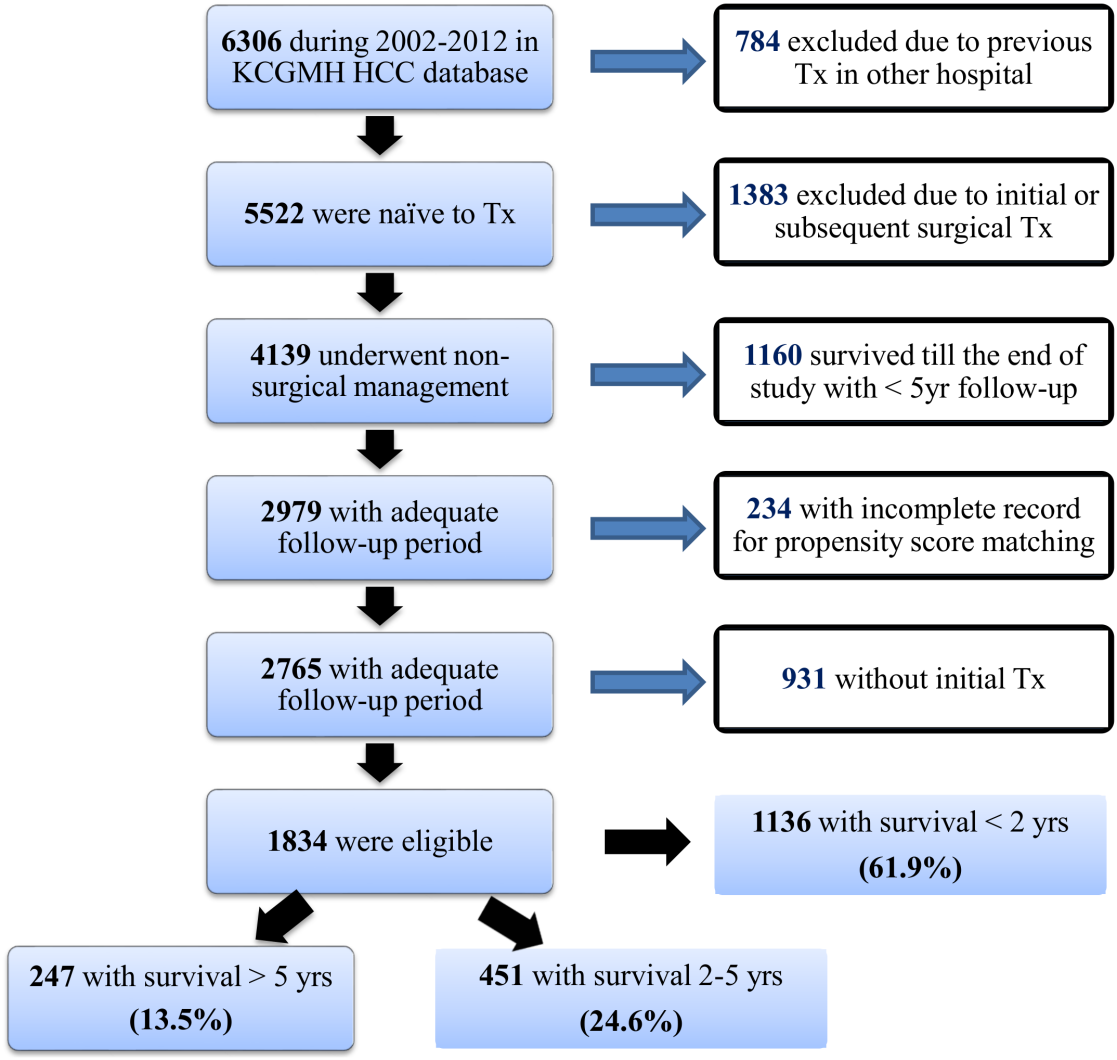
Treatment protocol in patients survived > 2 years (Tx: treatment).

Fig 1 illustrates the initial treatment modality and, in patients with incomplete first treatment or recurrence, the secondary treatment in both groups. (115 [25.5%] with RFA, 36 [8.0%] with PEI, 289 [64.1%] with TAE and 11 [2.4%] patients with RT or CT in group A, versus 93 [37.7%] with RFA, 25 [10.1%] with PEI, 126 [51.0%] with TAE and 3 [1.2%] patients with RT or CT in group B, respectively, *p* = 0.002). After the initial treatment, 645 patients (92.4%) had a complete first treatment. On other hand, 40 (8.9%) in group A versus 13 patients (5.3%) in group B had an incomplete treatment; and during subsequent follow-up, 237 (52.5%) in group A versus 167 patients (67.6%) in group B were recurrence free in the first year (*p*< 0.001).

Among the patients with subsequent recurrence and underwent secondary treatment, complete treatment were documented in 95 patients (31.3%) in group A versus 108 (57.4%) in group B, while 209 patients (78.7%) in group A and 80 (42.6%) in group B had incomplete treatment (*p*< 0.001). Viral hepatitis was found in 408 patients (90.5%) in group A and 226 patients (91.5%) in group B, but treatment were documented in 104 patients (25.5% among viral hepatitis) in group A and 93 patients in group B (41.2%, *p* < 0.001, Table 2).

**Table 2:** Initial treatment modality and treatment response of HCC, and anti-viral treatment status in Group A and Group B.

		Group A(n = 451, 64.6%)	Group B(n = 247,35.4%)	P value
Initial Tx	RFA	115 (25.5%)	93 (37.7%)	*0.002
	PEI	36 (8.0%)	25 (10.1%)	
	TAE	289 (64.1%)	126 (51.0%)	
	RT or CT	11 (2.4%)	3 (1.2%)	
1^st^Tx	Non recurrence within 1 year after complete Tx	237 (52.5%)	167 (67.6%)	*0.001
	Recurrence within 1 year after complete Tx	174 (38.6%)	67 (27.1%)	
	Incomplete Tx	40 (8.9%)	13 (5.3%)	
2^nd^Tx	Complete Tx	95 (31.3%)	108 (57.4%)	*<0.001
	Incomplete Tx	209 (68.7%)	80 (42.6%)	
Anti-viral Tx status	Tx Never Tx NBNC	104 (25.5%)304 (74.5%)43	93 (41.2%)133 (58.8%)21	<0.001

*:p< 0.01

Tx = treatment

### Propensity score match to adjust un-modifiable factors

We used propensity score match by serum albumin, bilirubin, and AFP levels to eliminate the impact of these un-modifiable baseline characteristics. After processing, both group A and group B had 223 patients, with identical baseline characteristics including liver function reserve, AFP levels, and BCLC stages that had been different in the raw data (Table 1). The treatment response of initial treatment modality, first complete treatment and one-year recurrence rate were all the same in both groups. However, in patients received secondary treatment, 58 patients (37.2%) in group A and 93 patients (55.0%) in group B had complete treatment (*p* = 0.002). In addition, the proportion of viral hepatitis was 204 (91.5%) in group A and 203 patients (91.0%) in group B and 57 (27.9%) in group A and 81 patients (39.9%) in group B underwent anti-viral treatment (*p* = 0.038, Table 3).We ran an univariate logistic regression and the results showed that recurrence in the first-year (odds ratio 1.64, *p* = 0.017), incomplete secondary treatment (odds ratio 2.07, *p* = 0.001), and absence of anti-viral treatment (odds ratio 1.71, *p* = 0.011) were associated with worse survival; the statistical significance was validated by further multivariate analysis (Table 4).

**Table 3:** Initial treatment modality and treatment response of HCC, and anti-viral treatment status in Group A and Group B after propensity score match.

		MatchedGroup A(n = 223)	MatchedGroup B(n = 223)	P value
Initial Tx	RFA	73 (32.7%)	82 (36.8%)	0.080
	PEI	10 (4.5%)	22 (9.9%)	
	TAE	137 (61.4%)	116 (52.0%)	
	RT or C/T	3 (1.3%)	3 (1.3%)	
1^st^ Tx	No recurrence within 1year after complete tx	128 (57.4%)	150 (67.3%)	0.052
	Recurrence within 1year after complete Tx	84 (37.7%)	60 (26.9%)	
	Incomplete Tx	11 (4.9%)	13 (5.8%)	
2^nd^ Tx	Complete tx	58 (37.2%)	93 (55.0%)	^#^0.002
	Incomplete tx	98 (62.8%)	76 (45.0%)	
Anti-viral Tx status	Tx	57 (27.9%)	81 (39.9%)	^#^0.038
	Never Tx	147 (72.1%)	122 (60.1%)	
	NBNC	19	20	

^#^:p< 0.01

Tx = treatment.

**Table 4:** Initial treatment modality and treatment response of HCC, and anti-viral treatment status in group A and group B after propensity score match.

		Univariate (A vs. B)	P value	Multivariate (A vs. B)	P value
Initial Tx	RFA	1			
	PEI	0.51(0.23–1.15)	0.104		
	TAE	1.33(0.89–1.98)	0.167		
	RT or C/T	1.12(0.22–5.74)	0.889		
1^st^ Tx	No recurrence in1 year after complete Tx	1		1	
	Recurrence in 1 year after complete Tx	1.64(1.09–2.46)^	^#^0.017	2.17(1.35–3.48)^	^#^0.017
	Incomplete Tx	0.99(0.43–2.29)^	0.984	1.12(0.46–2.72)^	0.984
2^nd^ Tx	Complete Tx	1		1	
	Incomplete Tx	2.07(1.33–3.22)	*0.001	2.01(1.27–3.17)^	*0.003
Anti-viral Tx status	Tx	1	0.011	1	^#^0.018
	Never Tx	1.71(1.13–2.59)^	0.410	1.68(1.09–2.59)^	0.388
	NBNC	1.35(0.66–2.76)^		1.38(0.66–2.89)^	

^#^:p < 0.05,

*:p < 0.01,

^: 95% confidence interval

Tx = treatment.

## Discussion

In HCC patients underwent non-surgical treatment, current international guidelines of HCC has used BCLC-based staging system to evaluate prognosis and guide initial therapeutic approach [12, 13]. Although widely accepted, this approach might have some limitations. On the one hand, the patients may receive different treatment modalities even in the same stage that confounds to prognosis; on the other hand, the disease course of HCC is dynamic; not only fluctuations of tumor burden but also alterations of liver functions impacts the choice of initial or subsequent treatment. As a result, staging systems based on initial parameters may not be so accurate as the patient survives longer, whereas modifiable prognostic factors may help prediction of outcome in patients had received treatment. In our study, the "conventional prognostic factors" like AFP level, Child-Pugh score and BCLC stages were still significant prognostic factors; however, after propensity score match, both complete locoregional control and anti-viral hepatitis treatment were found as independent significant modifiable prognostic factors.

Current concepts on management of HCC put much emphasis on surveillance and early diagnosis, which confers more curative treatment and improved prognosis [17–20], and RFA alone may be sufficient in patients who were not surgical candidates [21]. As to unresectable HCC, the importance of adequate tumor control were highlighted in two uncontrolled studies, in which patients received adjuvant locoregional therapy followed by orthostatic liver transplantation that pathologic complete response or ≥60 percent tumor necrosis were associated with better post-transplantation outcome [22, 23]. Although the results was similar, there was two major difference between the current and previous studies: firstly, our patients received non-surgical treatment only; Secondly, our study design was more strict; in addition to propensity score match to adjust the impacts of "un-modifiable factors" like liver function reserve and AFP, we only enrolled patients with survival > 2 years and adequate follow-up periods to avoid potential bias, since those with shorter survival more likely died from liver failure instead of HCC. Accordingly, the result may be more validated in the study.

In our study, recurrence within one year and complete secondary treatment were both significant prognostic factors. For surgical resected HCC, recurrence within the first year has been identified as an important prognostic factor in HCC [24], representing diffuse spread and difficult-to-treat tumors [25]. Although the data was lacking in non-surgical treated tumors, this observation may be true in patients received curative treatment as initial management. Besides, the non-ablative therapies including TAE, RT or CT might rarely cause a complete response of HCC, or reduce the tumor burden to make subsequent curative treatment possible, or even improved survival [26, 27]. Besides, for our patients with inadequate first treatment or recurrence, a complete secondary treatment still confer better survival; and the distribution of treatment modalities in group A and B were similar after propensity score match. Lastly, it seemed paradoxically that the recurrence rate was higher in group B than group A; however, this number included for all recurrence during the follow-up period; in other words, the longer survival as well as follow-up time resulted higher cumulative changes of delayed recurrence or new tumor occurrence, which highlighted the importance of complete secondary treatment in this group. Take together, it suggested that upon considering the treatment modality, the intervention carries best chance for locoregional control should be attempted as possible.

Another modifiable prognostic factor in this study was treatment to chronic hepatitis B and C. Previous data suggested active hepatitis was associated to worse prognosis [28], and tertiary prevention in HCC patients improved outcome and reduced tumor recurrence in both hepatitis B [29–31] and C [32–34]. However, most studies enrolled cases underwent surgical or curative treatment. Our findings indicated anti-viral treatment should not be overlooked in non-surgical treated patients.

To our best knowledge, this is the first study that addresses modifiable prognostic factors in HCC patients with non-surgical treatment. Admittedly, the study was limited by the retrospective nature and single-center origin of these patients. However, the large case numbers, long follow-up time and statistical methods aiming to eliminate baseline confounding factors might, at least in part, offset the possible biases. Secondly, despite trying to define the treatment modality and anti-viral treatment, there was inevitably some heterogeneity; besides, our definition of anti-viral therapy was broader then standard treatment protocol, thus the exact contribution of anti-viral agent to survival might be overrated. Therefore, carefully designed prospective studies might help elucidate to what extent anti-viral treatment provides survival benefit.

In conclusion, our study revealed complete treatment and anti-viral agents to chronic hepatitis were both independent modifiable prognostic factor of HCC patients had undergone non-surgical treatment. Based on these findings, timely treatment to achieve maximal locoregional control and anti-viral treatment should be provided as possible.

## Supporting Information

S1 Fig Patient enrollment in this study. (DOCX)

S2 Fig Kaplan-Meier survival curve in the patients received initial non-surgical treatment with adequate follow-up time. (DOCX)
