# Supplementary material for: Modifiable Prognostic Factors of Hepatocellular Carcinoma in Patients with Non-Surgical Treatment
Source: PLoS One. 2015 Dec 14;10(12):e0144893. doi: 10.1371/journal.pone.0144893 (PMC4685988; doi:10.1371/journal.pone.0144893)

**S2 Fig.** Kaplan-Meier survival curve in the patients received initial non-surgical treatment with adequate follow-up time.


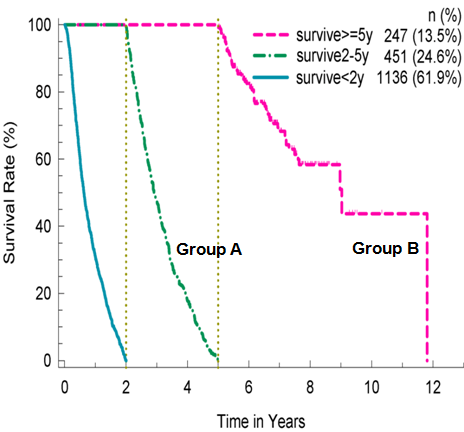

Supplement: S2 Fig — (DOCX) [file pone.0144893.s002.docx]
